# School wellbeing and psychological characteristics of online learning in families of children with and without hearing loss during the Covid‐19 pandemic

**DOI:** 10.1002/pits.22761

**Published:** 2022-07-13

**Authors:** Bianca Maria Serena Inguscio, Maria Nicastri, Ilaria Giallini, Antonio Greco, Fabio Babiloni, Giulia Cartocci, Patrizia Mancini

**Affiliations:** ^1^ Department of Sense Organs Sapienza University of Rome Rome Italy; ^2^ BrainSigns Srl Lungotevere Michelangelo Rome Italy; ^3^ Department of Molecular Medicine Sapienza University of Rome Rome Italy; ^4^ Department of Computer Science Hangzhou Dianzi University, Xiasha Higher Education Zone Hangzhou China

**Keywords:** hearing loss, online learning, school wellbeing

## Abstract

This study investigated the psychological characteristics of online learning on Italian students with and without hearing loss (HL) and on their parents, who were forced into isolation during the Covid‐19 pandemic. An online survey collected information on socio‐demographic data and opinions concerning online learning from 61 children (mean age 11; 25 males, 36 females), including 43 with HL and also from their parents; additionally, school wellbeing and anxiety were assessed. The results showed that, in both the student and parent groups, no significant effect of HL on school wellbeing and anxiety was found. Additionally, in parents, State Anxiety was significantly higher than Trait Anxiety, suggesting one possible impact of lockdown on psychological wellbeing. Differences due to HL were observed and discussed in correlation analyses. The Authors believe that this study is the first contribution to the psychological evaluation of the impact of online learning on families with hearing‐impaired children, from the perspective of a successful, inclusive didactic.

## Practitioner points

- Deafness globally did not impact school wellbeing during online learning for the Covid‐19 pandemic
- Teacher's relationship with deaf students as a protective factor against anxiety.
- The family environment was a protective factor during quarantine in Spring 2020.

## 1 INTRODUCTION

On 12 March 2020, World Health Organization (WHO) declared the coronavirus disease 2019 (Covid‐19) outbreak to be a pandemic (World Health Organization, 2020). Numerous countries have instituted large‐scale or national school closures to decelerate virus transmission by encouraging social distancing (Viner et al., 2020). As a result, in Spring 2020, many educational institutions decided to move from in‐person instruction to a remote learning model (Di Pietro et al., 2020). Online learning can be defined as “learning experienced through the internet/online computers in a synchronous classroom where students interact with instructors and other students and are not dependent on their physical location for participating in this online learning experience” (Singh & Thurman, 2019; p.302). Despite a plethora of technologies available for online education, complex student challenges can occur (Dhawan, 2020; Song et al., 2004). Researchers (R. T.‐H. Chen, 2010; O'Doherty et al., 2018) expressed their concerns about online learning and highlighted the main difficulties associated with creating an online learning community involving higher levels of social presence and engagement. In addition, scholars have concerns about social isolation, the lack of interactivity and participation and delayed or insubstantial amounts of feedback (Dong & Mertala, 2021; Khurana, 2016). It is evident that school closures during the coronavirus pandemic altered the daily lives of the students and their families and how children around the world were forced to become virtual‐school learners while their parents had to assume the role of pseudo‐teachers (Cohen & Kupferschmidt, 2020; Daniela et al., 2021; Garbe et al., 2020). These closures are likely to have damaged children's psychological and educational development and have caused loss of income and productivity in adults (Edmunds, 2020; Macartney et al.,^2020^). Millions of students were exposed to life changing impacts on their living environments, daily routines, and educational and social‐relational networks: critical contexts that promote mental health and resilience to traumatic events (Dalton et al., 2020; Danese et al., 2020). Considering the vital importance of routine school settings for the healthy development of children (Catalano et al., 2004; Segre et al., 2021) and the evidence that education “is health” (UNESCO, 2016), it is regrettably imaginable how this situation could have an amplified impact on children with sensory disabilities. In fact, previous studies have shown that deafness is already associated with significant heterogeneity in cognitive, social and emotional development (Holt et al., 2020; Kral & O'Donoghue, 2010). However, the psychological studies related to SARS Cov 2 and SARS Cov 1 primarily focused on the psychological status of typical people during the pandemic (Yang et al., 2021) and rarely, if at all, focused on deaf students (i.e., Alqraini & Alasim, 2021).

Studies have investigated the stress that parents experience associated with their HL children (Blank et al., 2020; Quittner et al., 2010), who may also face many other difficulties during distance learning courses because of the barriers their children face (Mantzikos & Lappa, 2020; McKeown & McKeown, 2019). Furthermore, a recent study conducted at the beginning of the coronavirus pandemic has highlighted the difficulties faced by the parents of children with special needs (SNs) who exert more effort when taking care of their children's learning and living conditions than that experienced by parents of typically developing children (Ren et al., 2020).

### 1.1 The current study

The primary aim of this study, titled COCLOVID (evaluation of the online **Di**dactic in deaf children with or without **COChL**ear implants and on their parents during the C**OVID**‐19 pandemic), was to investigate the psychological characteristics of online learning during the Covid‐19 lockdown on Italian students with and without hearing impairments and on their parents who were forced to isolate and undertake social distancing. In particular, we first investigated the possible influence of auditory features on Anxiety levels and School Wellbeing in the participants to try to answer the following research questions:

(1) Does having hearing difficulties or being the parent of such a child impact on School Wellbeing experienced during the period of online education?
(2) In the light of recent studies concerning the psychological and social effects of the Covid‐19 pandemic on young and adult populations, are there any differences in the psychological costs between children with or without hearing impairments and on the parents of healthy or hearing‐impaired children?
(3) Are there any significant correlations between psychological experiences and perceptions associated with School Wellbeing amongst students and their parents during the quarantine period?

To our knowledge, the experimental evaluation of School Wellbeing and Anxiety during online learning due to the Covid‐19 pandemic has received very little attention, especially concerning students with hearing impairments and the impacts upon their families. To date, we are not aware of any other study that has drawn attention to this specific topic.

## 2 METHODS

### 2.1 Study design and participants

COCLOVID is a study based on disseminating an ad hoc prepared online survey, managed through EUSurvey, a web platform promoted by the European Commission (2013). Questions were designed to collect and highlight the socio‐demographic data of parents and their children and on their opinions about school closures and online learning; the delivery of a standardized questionnaire enabled the assessment of the psychological dimensions of participants. The survey was broadcast through (i) email invitations to personal contacts and via healthcare professionals and their patients, (ii) social media channels. The survey was available from May to August 2020, taking approximately 30 min to complete. Participants were informed about the aims of the study and, before starting, electronic informed consent was requested from each parent and child. Participation in the study was voluntary, and therefore participants did not receive compensation for taking part in it. Data collection was conducted according to the principles outlined in the Helsinki Declaration of 1975, revised in 2000, and was approved by the Institutional Ethics Committee of Policlinico Umberto I, Rome, Italy  (No. 259/2020).

### 2.2 Outcomes

#### 2.2.1 General information

The survey questions collected socio‐demographic information (age, gender, education, occupation, region of origin, deafness, use of hearing aids or devices) and opinions about online learning.

#### 2.2.2 School wellbeing

The “Questionnaire on School Wellbeing” (QBS; Tobia & Marzocchi, 2015a), assessed children's (aged 8–13) wellbeing at school. It is based on a multidimensional concept of School Wellbeing that includes psychological, cognitive, and social components using a three‐perspective approach (indeed, the questionnaire investigates student, parent and teacher observations). In this study, we considered School Wellbeing from the perspective of students and parents.

The student version consists of 27 items and investigates the subjective school experience of male and female students attending primary school (3rd to 5th grade) and middle school (6th to 8th grade) by examining 5 QBS scale scores: (1). *Gratification obtained by school results—*GBS; (2)*. Relationship with classmates—*RWC; (3). *Relationship with teachers—*RWT; (4). *Emotional attitude towards school—*EA; (5). *Self‐efficacy—*SE and finally, a Total School Wellbeing—TOT score was obtained by combining the QBS scales scores. The parental version comprised of 36 items and five scales: (1). *Personal experience in relation to the child's difficulties—*PE; (2). *Evaluation of learning processes—*ELP; (3). *Child's emotional difficulties at school—*ED; (4). *Child's awareness of his/her difficulties—*CA; (5). *Relationship with teachers—*RWT.

With respect to the objectives of this study, only scales ELP, ED, RWT have been considered for parents. The “Multidimensional Self‐Esteem Test” (TMA, Bracken, 1993) is based on a hierarchical model of self‐esteem: it is comprised of six self‐esteem dimensions (*Personal*, *Skills*, *Emotional*, *School, Family*, and *Body*); the measure also includes a scale testing Total self‐esteem. The test consists of six groups of 25 items for each dimension explored, and each item requires one of 4 possible answers: absolutely true, true, not true, absolutely not true. The test provides scores on six rating scales corresponding to the six self‐esteem dimensions, and the Total self‐esteem‐related scores. The average scores for self‐esteem in the normative sample are between 85 and 115. In line with the aims of the study, we administered School and Family dimensions for 14–19‐year‐old students. The latter scales were related to the “General Self‐Efficacy scale” (GSE, Schwarzer & Jerusalem, 1995) to evaluate the individual's Self‐Efficacy. GSE is comprised of 10 items, scored on a 4‐point scale from 1 (*not at all true*) to 4 (exactly true); higher values indicate higher self‐efficacy. GSE psychometric characteristics have been extensively studied across several countries by Scholz et al. (2002), the Italian version (Sibilia et al., 1995) was used.

### 2.3 Anxiety

The “State‐Trait Anxiety Inventory for adults” (STAI‐Y, Spielberger et al., 1983; for Italian adaptation, see Pedrabissi & Santinello, 1989) was used with parents. It is comprised of separate self‐reporting scales for measuring *State* (S) and *Trait* anxiety. The S‐Anxiety scale (STAI Form Y‐1) consists of 20 statements that evaluate how respondents feel “right now, at this moment.” The T‐Anxiety scale (STAI Form Y‐2) consists of 20 statements that assess how people generally feel. Participants answered 40 items on a 4‐point Likert scale ranging from 0 (*not at all*) to 4 (*very much so*). The range score for each scale is 20–80, with the higher scores indicating greater anxiety.

The “Revised Children's Manifest Scale” (RCMAS‐2; Reynolds & Richmond, 1985; for Italian adaptation, see Reynolds et al., 2012) was used with students. RCMAS‐2 assesses both the degree and quality of anxiety experienced by children and adolescents (aged 6–19). It is a relatively brief instrument (49 items) suitable for administration in both clinical and educational settings. It is one of the most widely used questionnaires employed when researching and treating developmental anxiety (Gerard & Reynolds, 2014). Scores are provided for five distinct scales: *Physiological Anxiety* (PHY), *Concern* (CON), *Social Anxiety* (SOC), *Total Anxiety* (TOT), *Defensive Attitude* (DEF).

### 2.4 Statistical analysis

Descriptive statistics were performed to describe the sociodemographic and hearing characteristics, as well as opinions about online‐learning during Covid‐19 related aspects, in the parent and child populations. After checking the normality of each data distribution with both Shapiro–Wilk and Kolmogorov–Smirnov tests, independent *t* tests were used to compare the effects of Independent Variables (Auditory Condition [Normal Hearing {NH}/HL]; Gender [Male/Female]; School Grade [Elementary/Middle]) on Dependent Variables (TOTAL School Wellbeing; TOTAL School Self Esteem; TOTAL Family Self‐Esteem; TOTAL Self Efficacy and TOTAL Anxiety) in student groups and Trait Anxiety and State Anxiety, in parent groups. Subsequently, Factorial analysis of variance (ANOVAs) were performed for each questionnaire to investigate the influence of Independent Variables on the considered subscales. Independent Variables were: Auditory Condition (NH/HL), Hearing Groups (Unilateral cochlear implant user‐UCI, Bilateral cochlear implant user‐BCI, Bimodal hearing device user‐BIM, Hearing aid user‐HA, Normal hearing‐NH), Gender (M/F), School Grade (Primary/Middle), Parent Education (4 levels) and Family Income (4 levels); while the investigated questionnaire scales considered as Dependent variables were: QBS student scales (5 levels: GBS/RWC/RWT/EA/SE), QBS parent scales (3 levels: ELP/ED/RW) RCMAS‐2 scales (4 levels: PHY/CON/SOC/DIF), TMA scales (2 levels: SCH/FAM); STAI scales (State/Trait). Duncan's post hoc test (Duncan, 1955) was used to investigate statistically significant results of ANOVA tests; partial eta squared ${(\eta }_{p}^{2}$) (Cohen, 1973, 1988) were computed as measures of the effect size for each dependent variable. Finally, Pearson's Correlation Analysis (*r*) was performed to explore the correlation between study variables; *p* values of less than 0.05 were considered statistically significant.

## 3 RESULTS

### 3.1 Demographic characteristics

The characteristics of all participants are shown in Table S1. The survey was fully completed by 65 pairs (parent & child‐student) of participants. Due to the aims of the present research, the inclusion criteria adopted were for children aged between 8 and 19 years of age and who had no concurrent neuropsychiatric disorders; four pairs were therefore excluded from the study for declaration of diagnosed neuropsychiatric disorders. The final experimental population was composed of 51 mothers (83.607%, mean age 44.09 ± 6.23), 10 fathers (16.393%, mean age 49.66 ± 4.69), 25 sons (40.98%, mean age 12.33 ± 3.01) and 36 daughters (59.02%, mean age 12.19 ± 2.96). Amongst these 61 children, 43 of them were Hearing Device users divided into Unilateral Cochlear Implant users (UCI), Bilateral Cochlear Implant users (BCI), Bimodal Hearing Device users (BIM) and Hearing Aid (HA) users. Two student groups were created: (i) from 3rd to 8th grade = Junior Student group—JS (*N* = 45, mean age = 11 ± 1.73); (ii) attending high school = Senior Student group—SS (*N* = 17, mean age = 16.11 ± 1.21). According to hearing aid characteristics, in the JS group respectively, there were 7‐UCI, 10‐BCI, 8‐HA, 9‐BIM, and 11 NH; in the SS group, there were 6‐UCI, 1‐BCI, 2‐HA, and 7‐NH. Educational levels showed 9 (14.754%) parents with a Secondary School Diploma, 22 (36.066%) with a High School Diploma (13 years of study), 24 (39.344%) with a Bachelor's or Master's Degree, and 6 (9.836%) with a Post‐Graduate Degree. According to the responses to the questionnaire regarding online learning, overall parents (45.902%) reported a “*quiet”* level of concern about school closures with 4 parents of HL and 2 of NH children professed to be “*extremely*” concerned. For 54.098% of parents, online learning is “*quite useful*” whereas for 8 parents of HL children, compared to no parents of NH children, found it to be “*very useful*”. Overall students (49.180%) reported it to be “*quite*” enjoyable taking lessons online with 4 HL students, compared to no NH students, considering it to be “*extremely*” enjoyable taking online classes. The overall opinion about online lessons is that they were “*normal*” for 45.902% of students while 8 HL and 6 NH students considered it to be “*tiring*.” Only HL students (18.605%) described them as “*difficult to follow*.”

### 3.2 The general impact of conditions (NH/HL, M/F, middle/elementary) on anxiety and school wellbeing

The results of the *t* test conducted to compare Auditory Condition, Gender, and School Level variables are presented in Table 1. Considering all psychological assessments, there were no statistically significant differences between student and parent groups concerning all conditions (*p* > 0.05).

**Table 1:** Results of  *t* test for independent sample by groups

**(A)**	**µ HL group**	**µ NH group**	***t* value**	***df***	***p***	**Valid n HL**	**Valid n NH**	***SD* HL**	***SD* NH**
TOTAL School Wellbeing QBS (JS)	47	52.272	−1.299	43	0.200	34	11	11.88	11.055
TOTAL School self‐esteem scale—TMA (SS)	108.555	107.571	0.303	14	0.766	7	9	3.954	8.502
TOTAL Family self‐esteem scale—TMA(SS)	107.333	109.571	0.395	14	0.693	9	7	10.723	11.900
TOTAL Self‐efficacy scale (SS)	60.449	58.689	−1.000	14	0.189	9	7	2.902	1.928
TOTAL Anxiety RCMAS‐2 (all students)	48.323	43.222	−1.575	59	0.120	43	18	10.487	13.171
State Anxiety—STAI‐Y Test (Parents)	44.046	44.444	0.146	59	0.884	43	18	10.980	5.305
Trait Anxiety—STAI‐Y Test (Parents)	40.209	38.055	−0.933	59	0.354	43	18	8.730	6.786

**(B)**	**µ F group**	**µ M group**	***t* value**	***df***	***p***	**Valid *n* F**	**Valid *n* M**	***SD* F**	***SD* M**
TOTAL School Wellbeing QBS (JS)	50.444	45.055	1.525	43	0.134	27	18	12.911	9.276
TOTAL School self‐esteem TMA (SS)	109.000	107.000	0.623	14	0.543	9	7	5.612	7.257
TOTAL Family self‐esteem TMA (SS)	102.333	106.000	−0.380	14	0.709	9	7	23.590	10.503
TOTAL Self‐efficacy scale (SS)	39.888	41.285	−0.495	14	0.628	9	7	4.106	7.111
TOTAL Anxiety RCMAS‐2 (all students)	47.388	45.840	0.515	59	0.607	36	25	12.504	9.956
State Anxiety—STAI‐Y Test (Parents)	45.388	42.400	1.198	59	0.235	36	25	10.232	8.534
Trait Anxiety—STAI‐Y Test (Parents)	40.611	38.80	1.188	59	0.239	36	25	8.445	7.777

**(C)**	**µ Primary Students**	**µ Middle Students**	***t* value**	***df***	***p***	**Valid *n* primary students**	**Valid *n* middle students**	***SD* primary students**	***SD* middle students**
TOTAL School Wellbeing QBS (JS)	46.190	50.125	1.120	43	0.268	21	24	11.877	11.604
TOTAL Anxiety RCMS‐2 (JS)	48.190	46.375	−0.484	43	0.630	21	24	11.298	13.509
State Anxiety—STAI‐Y Test (Parents of JS)	45.333	45.375	0.013	43	0.988	21	24	10.233	9.863
Trait Anxiety—STAI‐Y Test (Parents of JS)	41.476	39.166	−0.941	43	0.351	21	24	8.394	8.052

*Note*: The table shows no differences between groups (A:HL/NH, B:M/F, C:Middle/Elementary) on Dependent variables (School Wellbeing, Anxiety).

Abbreviations: F, female; HL, hearing loss; M, male; NH, normal hearing; QBS, Questionnaire on School Wellbeing; *SD*, standard deviation.

### 3.3 School wellbeing

**Figure 1:**
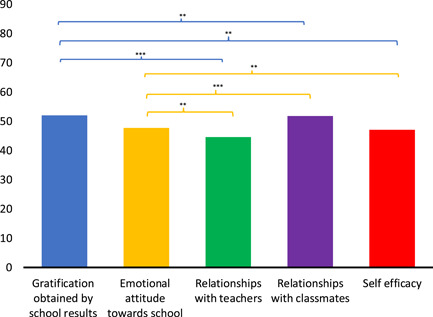
Junior Students School Wellbeing. The graph evidences the differences between QBS scales. Significant differences between QBS scales emerging from post hoc Duncan's test are indicated (*p ≤ 0.05; **p ≤ 0.01; ***p ≤ 0.001). Bars describe means, and error bars describe standard deviations

In the JS group, ANOVA results did not show an effect of the Auditory Condition variable on QBS scales (*F*_(1,43)_ = 0.992, *p* = 0.324, $\eta_{p}^{2}\;$= 0.022), but did show a significant difference within QBS scales (*F*_(4,172)_ = 3.432, *p* = 0.009 = 0.073). Post hoc analysis showed higher GBS and EA scores recorded rather than for the other three QBS scales (Figure 1).

**Figure 2:**
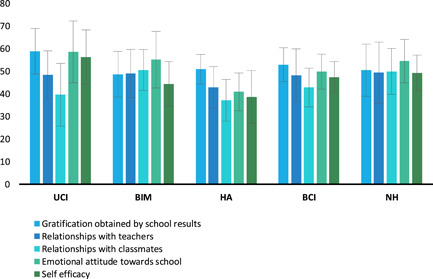
School wellbeing in Junior Students Hearing Groups. The graph shows the QBS scales values for JS Hearing Groups (for ANOVA's QBS scales × Groups post hoc analyses see Table S3). Bars describe means, and error bars describe standard deviations. ANOVA, analysis of variance; QBS, Questionnaire on School Wellbeing.

With regard to QBS scales, a nonsignificant effect within the variable Hearing Groups was found (*F*_(4,40)_ = 2.226, *p* = 0.083, $\eta_{p}^{2}\;$= 0.182); statistically significant differences amongst the QBS scales (*F*_(4,160)_ = 8.720, *p* < 0.001, $\eta_{p}^{2}\;$= 0.178) (Figure 1) and a significant interaction between QBS scales and Hearing Groups *(F*_(16,160)_ = 2.352, *p* = 0.003, $\eta_{p}^{2}\;$= 0.190) were found. Post hoc analysis showed differences between QBS scales similar to the ones found in Figure 1 and nonhomogeneity of QBS scores considering the effect of auditory characteristics (Figure 2). Several significant differences between and within Hearing Groups emerged from the analysis (Table S3). First, we observed numerous differences in QBS values between and within hearing loss groups (UCI, BCI, HA, BIM) but only slight differences between NH and HL groups and a total absence of differences within the NH group. A general observation concerning comparison amongst groups is that the HA group reported more statistically significant differences in the pairwise comparisons than with other Hearing Groups' QBS subscales scores.

**Figure 3:**
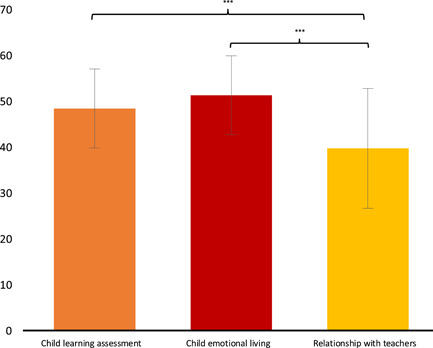
Parent's School Wellbeing. The graph shows significant differences between QBS parent's scales emerging from Duncan's Post hoc test (*p ≤ 0.05; **p ≤ 0.01; ***p ≤ 0.001). Bars describe means, and error bars describe standard deviations. QBS, Questionnaire on School Wellbeing.

QBS results in the parent group (of junior students), did not show any effect (*F*_(4,40)_ = 0.859, *p* = 0.496, $\eta_{p}^{2}\;$= 0.079), while there was a significant difference amongst QBS scales (*F*_(2,86)_ = 17.240, *p* < 0.001, $\eta_{p}^{2}\;$= 0.286), respectively. Post hoc analysis showed lower scores for the RWT scale than for ELP (*p* ≤ 0.001) and ED (*p* ≤ 0.001) (Figure 3). Moreover, QBS scales were not influenced by either Gender (of the child) (*F*_(1,43)_ = 0.042, *p* = 0.839, $\eta_{p}^{2}\;$= 0.000) or Income (*F*_(3,41)_ = 0.736, *p* = 0.536, $\eta_{p}^{2}\;$= 0.051).

**Figure 4:**
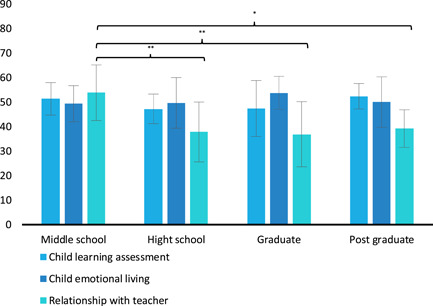
Parent's School Wellbeing for Education Level. The graph shows significant differences between QBS parent's scales and Education Level emerging from post hoc Duncan's test (*p ≤ 0.05; **p ≤ 0.01; ***p ≤ 0.001). Bars describe means, and error bars describe standard deviations. QBS, Questionnaire on School Wellbeing.

We observed, however, a significant effect of parents' Education Level on QBS scales (*F*_(6,82)_ = 2.626, *p* = 0.022, $\eta_{p}^{2}\;$= 0.161). Post hoc analysis showed that the only significant difference in the same scale between education levels emerged for RWT (i.e., the QBS scale that assesses the relationship and level of trust that parents have with their child's teachers): the values of parents with lower education levels were significantly higher in comparison to parents with High School (*p* = 0.005), Graduate (*p* = 0.003) and Postgraduate (*p* = 0.011) levels, respectively (Figure 4).

The results of ANOVA for the SS group showed that TMA scores were not influenced either by the factor Gender (*F*_(1,12)_ = 0.000, *p* = 0.992, $\eta_{p}^{2}\;$= 0.000) or by the factor Hearing (*F*_(1,12)_ = 0.5079, *p* = 0.489, $\eta_{p}^{2}\;$= 0.040).

### 3.4 Anxiety

**Figure 5:**
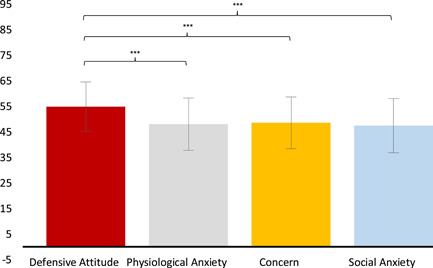
Anxiety in Student Group. The graph shows the significant differences between RCMAS‐2 scales emerging from Post hoc Duncan's test (*p ≤ 0.05; **p ≤ 0.01; ***p ≤ 0.001). Bars describe means, and error bars describe standard deviations

In all student participants, the overall results of factorial ANOVA analysis did not show any effect of either Auditory Condition (*F*_(1,57)_ = 0.243, *p* = 0.623, $\eta_{p}^{2}\;$= 0.004), or Gender (*F*_(1,57)_ = 0.182, *p* = 0.670, $\eta_{p}^{2}\;$= 0.003), or Hearing Groups (*F*_(4,56)_ = 0.416, *p* = 0.795, $\eta_{p}^{2}\;$= 0.028) whereas a strong significant difference was observed amongst RCMAS‐2 scales (*F*_(3,171)_ = 7.897, *p* ≤ 0.001, $\eta_{p}^{2}\;$= 0.121). The post hoc analysis showed significantly higher mean scores for the subscale *Defensive Attitude* than the other three scales (Figure 5).

**Figure 6:**
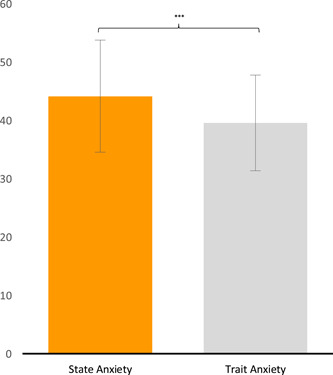
Anxiety in Parent Group. The graph shows the differences between State and Trait Anxiety STAI‐Y scales. Post hoc Duncan's test indicated (*p ≤ 0.05; **p ≤ 0.01; ***p ≤ 0.001). Bars describe means, and error bars describe standard deviations

In the Parent group, the results for the STAI‐Y scales × Auditory Condition × Gender factorial ANOVA analysis did not show any impact on Auditory Condition (*F*_(1.57)_ = 2.514, *p* = 0.118, $\eta_{p}^{2}\;$= 0.042] nor Gender (*F*_(1,57)_ = 0.449, *p* = 0.505, $\eta_{p}^{2}\;$= 0.007) on parent's anxiety levels but revealed significant differences between STAI‐Y scales (*F*_(1.57)_ = 25.929, *p* = 0.000, $\eta_{p}^{2}\;$= 0.312]. Post hoc analysis showed higher scores on State than on the Trait scale (*p* ≤ 0.001) (Figure 6).

In addition, further investigation into the effects of variables on anxiety levels did not show any significant variations for the parent Education Level (*F*_(3,57)_ = 2.078, *p* = 0.113, $\eta_{p}^{2}\;$= 0.098) nor for the family Income level (*F*_(3,57)_ = 0.468, *p* = 0.705, $\eta_{p}^{2}\;$= 0.024).

### 3.5 Factors correlated with anxiety, school wellbeing

The Pearson *r* coefficient was calculated between QBS scales and RCMAS‐2 scales to investigate the relationship between School Wellbeing and Anxiety variables amongst TOTAL, NH, and HL in the JS group (Table 2). The results showed no significant values between QBS scales and RCMAS‐2 scales in the Total (45) JS sample. However, considering HL and NH separately, results showed that in the HL group a negative correlation between *Defensive Attitude* and RWT (*r* = −0.35, *p* < 0.05) was absent in NH group. In the latter group strong positive correlations were noted between *Defensive Attitude* and RWC (*r* = 0.85, *p* < 0.05) and between *Defensive Attitude* and SE (*r* = 0.72, *p* < 0.05). In the Parent group the results of the correlation analysis between the answers to qualitative items within the survey and, respectively, QBS (parent) scales and STAI‐Y scales are shown in Table 3 **(A** and **B)**. As inferred from the results, there was a strong negative correlation between State anxiety and the level of education of the NH parent group (*r* = −0.66, *p* < 0.05) (Table 3 [**B**]). Moreover, we observed a relationship between overall Parent and Student anxiety variables (0.34 ≤ *r* ≤ 0.64, *p* < 0.05), as shown in Table 4.

**Table 2:** Relationships between school wellbeing (QBS) and anxiety (RCMAS‐2) in total student population

RCMAS‐2	Defensive Attitude			Total Anxiety			Physiological Anxiety			Concern			Social Anxiety		
QBS scales (Children)	TOT JS	NH JS	HL JS	TOT JS	NH JS	HL JS	TOT JS	NH JS	HL JS	TOT JS	NH JS	HL JS	TOT JS	NH JS	HL JS
GBS	−0.09	0.1	−0.14	0.03	0.35	−0.18	−0.13	−0.24	−0.12	−0.26	−0.42	−0.22	−0.12	−0.12	−0.13
RWT	−0.23	0.11	−0.35*	0.14	0.29	0.1	0.05	0.11	0.04	0.1	−0.03	0.16	0.07	0.09	0.07
RWC	0.14	0.85*	−0.02	−0.05	−0.15	0.09	0.05	0.18	0.06	0.08	0.11	0.09	0.16	0.57	0.07
EA	0.19	0.37	0.15	−0.18	−0.33	−0.1	−0.08	0.01	−0.08	−0.08	0.15	−0.12	0.01	0.34	−0.07
SE	0.22	0.72*	0.13	−0.16	−0.99	−0.15	−0.03	0.26	−0.07	−0.18	−0.14	−0.18	−0.07	0.2	−0.13
TSW	0.04	0.42	−0.05	−0.12	−0.1	−0.08	−0.08	−0.03	−0.07	−0.12	−0.21	−0.08	0	0.2	−0.05

*Note*: In bold, * = correlation (*r*) significant at *p* < 0.05.

Abbreviations: EA, emotional attitude towards school; GBS, gratification obtained by school results; QBS, Questionnaire on School Wellbeing; RWC, relationship with classmates; RWT, relationship with teachers; SE, self efficacy; TSW, total school wellbeing.

**Table 3:** Relationships between Qualitative Items and School Wellbeing (QBS) and Anxiety (STAY‐Y) in Parents (P) of all Students and Junior Students (JS).

*Items*		*Income*			*Instruction*			*Internet connection at home*			*Opinion about online learning*			*Is your child wasting time?*		
***5 A QBS scales*** a	*P_TOT JS*	*P_NH _JS*	*P_HL _JS*	*P_TOT_ JS*	*P_NH_ JS*	*P_HL_ JS*	*P_TOT_ JS*	*P_NH_ JS*	*P_HL_ JS*	*P_TOT_ JS*	*P_NH _JS*	*P_HL _JS*	*P_TOT_ JS*	*P_NH_JS*	*P_HL _JS*	*P_TOT_ JS*
***ELP***	0.2 4	0.38	0.25	0.01	0.08	0.02	0	0.01	0.01	0.21	0.29	0.19	0.2	0.17	0.24	−0.12
***ED***	0.1 5	0.27	0.25*	0.13	−0.26	0.17	−0.06	0.08	−0.1	0.13	0	0.16	0.03	−0.51	0.14	−0.21
***RWT***	1	0.06	0.02	−0.28	−0.15	−0.3	0.05	−0.36	0.17	0.27	0.21	0.29	0.17	0.74*	0.04	−0.07
***5 B STAI‐Y***	*P_TOT*	*P_NH*	*P_HL*	*P_TOT*	*P_NH*	*P_HL*	*P_TOT*	*P_NH*	*P_HL*	*P_TOT*	*P_NH*	*P_HL*	*P_TOT*	*P_NH*	*P_HL*	*P_TOT*
***Sate***	0.0 2	−0.1	0.04	0.09	− 0.66*	0.20	−0.01	0.54*	0.11	−0.27*	−0.3	− 0.28	−0.09	0.07	−0.13	0.25
***Trait***	0.0 6	0.001	0.17	0.06	−0.45	0.21	−0.25	0.59*	−0.11	−0.16	−0.12	− 0.18	−0.04	0.24	−0.11	0.16

*Items*		*Is it beneficial to take classes online?*		*Presence of parent during online lessons*				*Need of Additional tools*			*Concern about school closure*	
***5 A QBS scales*** a	*P_TOT JS*	*P_NH _JS*	*P_HL_JS*	*P_TOT_ JS*	*P_NH_JS*	*P_HL_JS*	*P_TOT_ JS*	*P_NH_JS*	*P_HL_JS*	*P_TOT_JS*	*P_NH_JS*	*P_HL_JS*
***ELP***	0.2 4	−0.14	−0.14	−0.39*	−0.28	0.54*	0	−0.57	0.28	−0.29	−0.31	−0.28
***ED***	0.1 5	−0.3	−0.17	−0.31*	−0.53	−0.25	0.15	−0.35	0.28	−0.13	−0.47	−0.1
***RWT***	1	0.4	−0.19	−0.02	0.5	−0.13	−0.08	0.2	−0.15	−0.21	−0.17	−0.21
***5 B STAI‐Y***	*P_TOT*	*P_NH*	*P_ HL*	*P_TOT*	*P_NH*	*P_HL*	*P_TOT*	*P_NH*	*P_HL*	*P_TOT*	*P_NH*	*P_HL*
***Sate***	0.0 2	0.38	0.38	0.34*	0.12	0.38*	−0.02	−0.1	−0.19	0.38	−0.54*	0.4*
***Trait***	0.0 6	0.37	0.2	0.21	0.11	0.2	−0.06	−0.23	−0.2	0.25	−0.59*	0.23

In red

* =correlation (r) is significant at p < 0.05 level.

^a^ =abbreviations of QBS parent's scales:

ELP‐Evaluation of learning processes; ED‐Child's emotional difficulties at school; RWT‐Relationship with teachers.

**Table 4:** Relationships between anxiety in parent (STAI‐Y) and anxiety in student (RCMAS‐2)

RCMAS‐2	Defensive Attitude			Total Anxiety			Physiological Anxiety			Concern			Social Anxiety		
STAI‐Y	TOT POP	TOT NH	TOT HL	TOT POP	TOT NH	TOT HL	TOT POP	TOT NH	TOT HL	TOT POP	TOT NH	TOT HL	TOT POP	TOT NH	TOT HL
State Anxiety	−0.03	0.58*	−0.13	0.34*	0.41	0.37*	0.43*	0.60*	0.41*	0.35*	0.67*	0.31*	0.30*	0.54*	0.26
Trait Anxiety	−0.04	0.45	−0.18	0.37*	0.39	0.35*	0.39*	0.39	0.39*	0.46*	0.65*	0.39*	0.25	0.58*	0.21

*Note*: In bold, * = correlation (*r*) significant at *p* < 0.05 level.

## 4 DISCUSSION

### 4.1 School wellbeing

#### 4.1.1 Students

Overall, the student group presents values on QBS scales in the average when compared to the reference norms (Tobia & Marzocchi, 2015a) (Figure 1). The direct comparison of NH and HL Young Students on the TOT‐QBS scale does not reveal significant differences (Table 1 [**A**]): the deafness factor does not affect the Total School Wellbeing results in the studied sample. This outcome may not be in line with research showing that deaf children experience disadvantages in the educational system (Berry, 2017). However, it is in line with studies showing that most cochlear implanted children in mainstream schools seem to have a positive attitude towards self‐esteem and confidence (Choi et al., 2016). Moreover, as Rotsika et al. (2011) suggested, struggling children (like deaf children) may underestimate their problems to protect themselves from the pain of facing their difficulties. This assumption could be supported by the fact that compared to their NH peers, of whom only 5.556% consider online lessons “*funny*,” 0.00% to be “*difficult*” and 16.667% “interesting.” Whereas for HL students, online classes are considered by 11.628% to be “*funny*,” 18.605% to be “*difficult to follow*” but for 18.605% of them to be “*interesting*” (Table S1).

Even considering the five QBS scales, we have seen that no significant differences between NH/HL children emerged. Ayfer and Ocakçi (2012) found that children with HL, compared to NH using the Kid_KINDL scale for the evaluation of quality of life (for details, see Lin et al., 2014), had significantly lower scores in terms of emotional, family, School Wellbeing and self‐esteem results in the scales of the questionnaire. Furthermore, another study (Yigider et al., 2020) reported that the leading quality of life scores of children with HL, according to the same Kid‐ KINDL scale, were significantly lower than for healthy children. However, the Authors did not find differences between NH and HL groups in terms of family and school scales, but the HL group had significantly lower scores in emotional wellbeing and social relationships (psychological variables also present in QBS). Therefore, the above‐cited literature shows that hearing loss may dramatically reduce quality of life in the pediatric population. These observations give rise to further important considerations.

Self‐Esteem is a crucial component in human beings' psychological wellbeing and life satisfaction (Borowiec et al., 2019; Rosenberg, 1965). In addition, Self‐Esteem, as a component of emotional wellbeing is one of the dimensions of quality of life (Knox & Muros, 2017). Moreover, a global report on disability (World Health Organization‐WHO, 2011) highlighted the need to undertake research and make interventions to improve quality of life and its dimensions among people with disabilities. In this perspective, studies concerning the Self‐Esteem of HL people observed a lower Self‐Esteem level among HL children compared with their peers (Borowiec et al., 2019; Lesar & Smrtnik Vituli, 2014). Studies also show that the leading causes of lower Self‐Esteem in HL children are difficulties in communication and the inability to form peer relationships (Fellinger et al., 2012). Moreover, Self‐Efficacy is predictive of higher Self‐Esteem (Stroiney Hermann, 2005) and has been applied to such diverse areas as school achievement and emotional disorders (Schwarzer et al., 1997). According to Bandura (1997), Self‐Efficacy makes a difference in how people feel, think and act: low Self‐Efficacy is associated with low Self‐Esteem: both Self‐Esteem and Self‐Efficacy, when measured, showed significant positive intercorrelations (Lane et al., 2004). Our results do not show differences between NH and HL children in QBS SE. However, in the light of the literature, we should have expected significantly lower scores in HL students on the QBS scales, specifically concerning SE, EA, and GBS scales. To explain this lack of difference between HL and NH, we can advance two hypotheses:

(i). During lockdown, the family environment has been a strong protective factor for School Wellbeing perceived by the HL child allowing this group to have QBS scores not significantly different from the NH group. This hypothesis could be verified by repeating the study after the return to a face‐to‐face didactic setting.
(ii). Given the different auditory features in HL students, it could be assumed that the nonhomogeneity of deafness characteristics in the HL group could increase their overall mean scores resulting in the loss of any differences when compared to the NH group. Whilst exploring this aspect, we performed a further analysis of the Hearing Groups variable (Figure 2). Results revealed significant differences between students with different hearing devices: the characteristics of deafness in the HL groups could significantly increase the mean scores of School Wellbeing. Such evidence suggests the need for further investigation and is probably explained by a high heterogeneity due to the HA clinical condition. More specifically, the evidence of a nonhomogeneity of the QBS scales' scores both *within* and *between* HL subgroups and the homogeneity found *within* the NH group (Table S3), could be one of the causes of the lack of significant differences in QBS scores between NH and HL. Based on this observation, analysis of future data possibly taken in a regular didactic setting could refine and validate whether the audiological characteristics of HL students could play a decisive role in this lack of statistical variation.

A variable that reportedly influences not only School Wellbeing but also its potential predictors is Gender (Løhre et al., 2010) and consequently we investigated the impact of the Gender variable on TOTAL School Wellbeing. The main finding of an interesting study was that girls within each school level rated School Wellbeing more positively than boys even though the latter had fewer symptoms than girls (Konu & Lintonen, 2006). Similarly, Hascher and Hagenauer (2011) reported that female students had a more positive attitude towards school and greater enjoyment of it, but that they also had more somatic complaints and worries than male students. The latter were more distressed than females, but no significant gender effect was observed on depressive symptoms (Correia & Dalbert, 2007; Peter et al., 2013). In contrast, it has been reported that girls generally have higher negative affect scores and lower positive affect scores than boys (Clark & Watson, 1991), but at the same time, girls are more motivated to study than boys (Alivernini et al., 2018; Grouzet et al., 2006). Others assert that gender is a relevant factor in students' feelings at school: being female negatively affects it (Alivernini et al., 2018). Our initial analysis to assess whether Gender influences the Total School Wellbeing variable does not reveal any significant difference between M and F groups (Table 1 [**B**]). Although this result may be at odds with parts of the literature, one might speculate, as proposed by Savoye et al. (2015), that other factors related to wellbeing could interact with gender differences in School Wellbeing within our junior student sample.

We could suppose that during the pandemic period characterized by online teaching, neither the Gender factor nor Hearing (NH/HL) influenced the School Wellbeing of the students neither globally (TOT‐QBS) nor at the level of the internal components (QBS scales). This observation can be considered partially in accord with the study on School Wellbeing in children with special educational needs‐SEN (Tobia & Marzocchi, 2015b) that highlighted higher scores for control subjects in the scales of GBS, RWT, and SE, while no significant differences emerged between RWC and EA. Other findings have shown that children with SEN tend to have lower subjective wellbeing levels than children without SEN when talking about their schools (Barnes & Harrison, 2017).

School wellbeing also appeared to be significantly different between schools and between classes in the same school (Holfve‐Sabel, 2014). For example, a study found that students in 4th‐6th grades experienced better school conditions, social relationships, and self‐fulfillment than students in 7th−12th grades (Konu & Lintonen, 2006). Comparing Middle and Elementary students, our data did not show significant differences in Total School Wellbeing (Table 1 [**C**]). This result contrasts with a QBS study that supported a higher level of School Wellbeing for the younger students (Tobia et al., 2019) but is in line with a study that compared associations across all school grades (Løhre et al., 2010), where a substantial effect of scholar grade on School Wellbeing variables was not observed.

Moreover, the scales used to evaluate School Wellbeing in the senior student populations were not found to be influenced by either Auditory Condition or Gender factors. However, this lack of significant effect of the factor Gender on TMA‐School and Family scales, is in line with the Italian validation of the TMA questionnaire, which shows significant differences for several scales but not for the two used in the present study (Bergamini & Pedrabissi, 2003).

#### 4.1.2 Parents

With regard to the QBS scales used to evaluate children's School Wellbeing as perceived by their parents, no significant differences emerged amongst parents of NH or HL children: the deafness factor does not seem to influence parents' assessment. Significant differences emerged in the Parent sample amongst the scales: the score on *Relationship with teachers* is significantly lower than scores on ELP and ED scales (Figure 3). These results resonate with the idea that for online‐learning during Covid‐19 quarantine, overall parents seem to have given lower ratings (but still in a medium average compared to the QBS reference norms in Tobia & Marzocchi, 2015a) to the relationship that their child had with their teacher than that of the assessment of learning and the emotional experience of the child. It may be that this difference is due to the lack of direct contact between students and teachers. Our results differ in part from those of a study by the same authors just mentioned (2015) that showed, in the comparison between SNs and healthy children and their parents, significant differences in all QBS parent's scales, except for the RWT scale. This latter result is in line with our study and seems to support the hypothesis that the parent's evaluation of the relationship with their teacher is not a variable related to the child's specific needs.

We did not find significant differences in the interaction between QBS parent scales and the Gender of the child. This result differs from data reported in Tobia & Marzocchi, 2015b; which observed that parents gave a more significant evaluation of School Wellbeing in the case of a daughter and evaluated them as more competent in learning processes and managing school material difficulties. However, statistical differences between the scales are not reported in the cited study, and therefore an evidence‐based assessment cannot be made here. Our results, indeed, are not in line with the evidence indicating that females are more competent in school (e.g., Berchialla et al., 2011; Hyde & Linn, 2006; Pomerantz et al., 2002; Spelke, 2005) but may be partially ascribed to the current pandemic situation that could result in the reduction of the effect of the intervening variable Gender of the child in assessing the child's perceived academic well‐being by the parent.

The Education Level of parents seems to influence the scores of the QBS scale (Figure 4). More specifically, the most substantial difference emerges for the RWT scale: parents with an inferior level of education (until middle school) reported higher scores on the evaluation of the teacher and so we can speculate that they were more involved in this relationship during the quarantine period. As evidenced by Mantovani and Gasperoni (2017), parental involvement varies due to various factors, which may also act ambivalently. For example, higher levels of education are positively associated with school participation (Crozier, 1999; Lee & Bowen, 2006; Peña, 2000; Potvin et al., 1999); but, conversely, parents with college degrees may be less likely to participate due to lack of time (Bæck, 2010).

However, this consideration needs more data to come to an informed position which would deviate from the purpose of this study.

### 4.2 Anxiety

Nowadays, anxiety disorders (American Psychiatric Association, 2013) are a major worldwide health problem with sizeable psychological, social, and economic costs (Beddington et al., 2008). During childhood and adolescence, anxiety phenomena are highly prevalent (e.g., Broeren & Muris, 2009; Craske et al., 2013), affecting around 10% of children and 20% of adolescents (Essau et al., 2012). They can negatively interfere with general wellbeing, social life, academic performance and development of social skills (Kessler et al., 1995; Pine et al., 1998), independently of culture (Crawford & Manassis, 2001). When compared to ordinary hearing people, children and adolescents with hearing impairment have been found to suffer more from behavioral and emotional problems, including social anxiety (Hindley, 2005). Recent studies on the Italian population indicated that the Covid‐19 pandemic appears to be a risk factor for higher levels of anxiety in younger and older adults (e.g., Casagrande et al., 2020; Germani et al., 2020; Rossi et al., 2020). According to these studies, the assessment of the anxiety state of participants resulted in it being extremely important for a complete evaluation of School Wellbeing, especially in children with sensory disabilities.

### 4.3 Students

Overall, the mean values for all types of anxiety investigated within the RCMAS‐2 questionnaire amongst the entire student group and subdivisions in NH/HL, are “*not particularly problematic*” as reported in the Italian norms.

(Reynolds et al., 2012). Moreover, anxiety symptomatology does not differ significantly by hearing or gender of the student. (Table 1 [**A**]). Regarding deafness, our results would seem to be at odds with the literature regarding SNs students (e.g., learning disability‐LD). In fact, children with LD have been found to show higher levels of trait anxiety (M. Bender, 1993; W. N. Bender & Wall, 1994) and a high rate of anxiety disorder (Beitchman et al., 1996); a meta‐analysis confirms that students with LD experience higher levels of anxious symptomatology than do their non‐LD peers (Nelson & Harwood, 2011). Furthermore, it is well known that auditory processing has a crucial role in language development (Moeller et al., 2007; Bailey & Snowling, 2002) and that school‐aged children who are hearing impaired are five times more likely to suffer from emotional disturbance (Wolters et al., 2012). Even slight or mild hearing impairment can result in negative consequences in the psychological domain, and there is a significant relationship between delayed language, anxiety and emotional‐related problems (Azab et al., 2015). In the light of the literature, we can assume that our sample has undifferentiated anxiety symptomatology based on difficulties related to deafness, and protective interventions towards children (HL) most at risk during the quarantine. This speculation may be further corroborated by comparison with the results of the data possibly collected during the return to face‐to‐face teaching.

The significantly higher score found for the *Defensive attitude* RCMAS‐2 scale across all student participants compared to other scales (Figure 5) suggests that students are unwilling to admit common failings or have attempted to give a very positive self‐image in a *naïve* or immature way. In fact, the *Defensive attitude* scale, containing items such as “I never get angry,” “I like everyone I know,” “I am always kind” is often used as an indicator of social desirability (Dadds et al., 1998) or/and defensiveness (Joiner et al., 1996). In some cases, high values express an excessive need for social desirability or acceptance (Reynolds & Richmond, 2008). As shown previously (Figure 5), *Defensive attitude* scale scores in our population are in a “not particularly problematic” range (see Reynolds et al., 2012 for normative standard). However, the mean score is significantly higher than that of other scales, suggesting a general lack of self‐observation and severe aversion to self‐observation in students. This evidence could imply a general tendency towards closure in our studied student sample during the lockdown period. Did the quarantine situation increase the *Defensive attitude* of students during online education? Moreover, the correlation analysis results showed no significant values between QBS scales and RCMAS‐2 scales in the Total (45) Junior student group. However, considering HL and NH separately, results showed, in the HL group, a negative correlation between *Defensive Attitude (RCMAS)* and RWT (QBS) (*r* = −0.35, *p* < 0.05) not present in the NH group. Whereas, the latter group revealed a strong positive correlation between *Defensive Attitude* and RWC (*r* = 0.85, *p* < 0.05) and between *Defensive Attitude* and SE (*r* = 0.72, *p* < 0.05). It is well known that the teacher‐student relationship is predictive of classroom wellbeing (Murray & Pianta, 2007; Spilt et al., 2011; Wolters et al., 2012). Moreover, the relationship with the teacher is potentially even more significant for the wellbeing of students with disabilities (Murray & Greenberg, 2001; Murray & Pianta, 2007). For deaf children in special education schools, a more positive relationship with the teacher increases wellbeing in school in both Grades 6 and 7 (Wolters et al., 2012). We can hypothesize that the negative correlation between *Defensive attitude* and RWT may represent a synergistic element in the wellbeing of the HL child during the quarantine school isolation period. Regarding the correlations in the NH group between *Defensive attitude* and RWC and SE, respectively, our results appear to be in line with the literature that has shown how children and adolescents rely heavily on the evaluation of others for self‐assessment. From these assertions, regarding our results, one could possibly advance the hypothesis that during the pandemic, the insecurity due to the lack of physical and nonvirtual regularity of the relationship with peers has increased in the NH population a defensive attitude in an attempt to preserve the quality of their relationship with peers and the maintenance of self‐esteem. Despite previous evidence (P. K. Bender et al., 2012), in the present study sample, Gender did not significantly influence clinical anxiety features (Table 1 [**B**]). This lack of difference could be because anxiety was assessed via self‐ reporting measures (online survey). In fact, although this methodology is common in the literature (e.g., Garnefski et al., 2005; Martin & Dahlen, 2005), self‐reporting measures may cause some bias in the way they require respondents to report on their behavior. It is worth noting that, on average, girls obtained higher scores in total anxiety than males (see Table 1 [**B**]) as reported in the normative groups (Reynolds et al., 2012).

Furthermore, the higher value of *Defensive attitude* compared to the other RCMAS‐2 scales, supports the idea that the student groups (regardless of Gender and Hearing factors) concealed some anxiety symptoms, at least during lockdown. Will the strong defensive attitude persist even after the restoration of face‐to‐face teaching? Did quarantine increase defensive attitudes in NH or HL children more than with face‐to‐face teaching? Once again, we may possibly answer these intriguing questions in a future study involving regular teaching conditions.

### 4.4 Parents

With regard to the assessment of the overall parent population, low symptoms of Trait anxiety and mild symptoms of State anxiety are observed in respect of the Italian normative standard (Spielberger, 2018). It is possible to suggest that, in line with recent studies (Marchetti et al., 2020; Mazza et al., 2020; Prete et al., 2020), the general population's level of anxiety has risen due to Covid‐19 fear and uncertainty.

We observed a significant global difference between Trait and State anxiety scores in parents: the latter are higher (Figure 6). According to Spielberger (1966; 1979), State anxiety reflects the transitory emotional state of human reactions directly related to adverse situations in a specific moment of the life. In contrast, Trait anxiety refers to a trait of personality, describing individual differences related to the predisposition to respond anxiously to certain situations. Our results show ho1w the adverse conditions faced by these parents during lockdown due to Covid‐19, significantly influenced their levels of anxiety. Moreover, this evidence is in line with recent studies that reported a high level of anxiety among Italian adults during the pandemic (Casagrande et al., 2020; Cellini et al., 2020; Rossi et al., 2020), supporting the hypothesis that during quarantine, Italian parents evaluated the pandemic as severe, showing a realistic perception of the critical situation. In keeping with our research proposal, we conducted additional analysis to understand whether other factors, in addition to the quarantine period, influenced the anxious parental state.

No differences emerged between parents with a HL or NH child. Studies posit that the levels of parental anxiety affect the physical and mental development of children (e.g., Kennedy et al., 2009; Ramchandani et al., 2005; Sprang & Silman, 2013). It is therefore sadly imaginable how parents of SN children (and so HL children) who have to undertake the tasks of childcare, training, rehabilitation and learning during the pandemic would pose an immense challenge for them.

Exploring parental anxiety under stress and the corresponding influencing factors prevalent during Covid‐19 will help healthcare professionals to provide targeted guidance and assistance. From this perspective, it is reasonable to believe that parents of HL children will have been more stressed than usual during the pandemic because they would have had to take care of their children's education and overall wellbeing, which eventually leads to an increase in anxiety state levels.

Our results are partially in line with a fascinating recent study (Ren et al., 2020) about the state anxiety of parents of SN children during Covid‐19 that shows significant differences in the results amongst parents. Authors found that the state anxiety score was significantly higher in parents of children with autism than in parents with visual impairment, while they have not observed any differences amongst parents of autistic, intellectual, and hearing‐impaired children. However, the study design of Ren and colleagues (2020), did not include a group of parents of healthy children, and so no comparison with a control group has been assessed. Therefore, in an evaluative comparison between our results and those of Ren (2020) it is not possible to test whether additional factors could contribute to any lack of significant differences between HL parents and NH ones: for example, in the identification of protective factors for state anxiety in parents with SN children or the presence of risk factors in parents with healthy children. With respect to the question of whether there is the existence or absence of additional factors that could justify the lack of significant differences between a parent of a HL student and those of NH students, we have further deepened the analysis with additional variables (Gender, Income, Education).

The Gender of the child does not affect the level of parental anxiety. Some studies on younger children found that same‐gender parent‐child dyads demonstrate a strong relationship between parent and child psychopathology (e.g., Ensminger et al., 2003; Wahl & Metzner, 2012), whereas studies on emerging adults have indicated that opposite gender parent‐child dyads tend to have the strongest associations between parent and child psychopathology (McKinney & Brown, & Malkin, 2018; McKinney & Kwan, 2018; Walker & McKinney, 2015). However, although the literature emphasizes the correlation between mental disorder within parent‐child dyads, few studies have delved into the differences in parental state anxiety based on child gender. As noted for the Auditory Condition factor, we did not find a significant effect due to the Gender of the child on parental State and Trait anxiety. However, we observed significant correlations between child anxiety and parent anxiety in both NH and HL populations (Table 4). It is interesting to note how the Total anxiety in students is correlated with both State (*r* = 0.37, *p* < 0.05) and Trait anxiety of parents (*r* = 0.35, *p* < 0.05) in the HL group and not in the NH group. Moreover, for all RCMAS‐2 scales, positive correlations with State and Trait anxiety are observed both in NH and HL groups but with higher values in NH than in the HL group. Thus, although State and Trait anxiety may be concomitant factors with the child's Total anxiety, the components of this psychic condition (in particular *Defensive attitude and Concern*) seem to be most strongly correlated in the NH group (0.45 ≥ *rNH* ≤ 0.67, 0.35 ≥* rHL* ≤ 0.41; *p *< 0.05). We can speculate that the tendency to conceal certain aspects of oneself and appear differently from how one is, would seem to be more aligned within the NH population to parental anxiety status during quarantine for Covid‐19.

Studies investigating the risk factors for anxiety caused by the Covid‐19 outbreak reported that anxiety or depression were associated with loss of income due to the pandemic (Hyland et al., 2020). Another study found no significant association between occupation, income, and anxiety during this challenging period (Blbas et al., 2020). Moreover, as observed in a recent review by Brooks et al. (2020), financial loss can be a severe problem during the pandemic. Authors reported that economic loss due to quarantine created serious socioeconomic distress (Pellecchia et al., 2015) and was found to be a risk factor for symptoms of psychological disorders (Mihashi et al., 2009) and resulted in both anger and anxiety several months after quarantine (Jeong et al., 2016) Recently, L. Chen et al. (2021) showed a trend of negative correlation between income levels and STAI‐Y scores, finding also that the incidence of severe anxiety and STAI‐Y scores in low‐income groups significantly increased during the quarantine period. However, to date, few studies (if any) have focussed on the factors that may affect the anxiety of quarantined parents with HL children. Although pandemic diseases, as seen previously, were found to have been associated with high levels of anxiety as recorded in recent literature, the mechanism underlying specific processes is still unclear, especially, in disability affected populations. For example, Ren et al. (2020) found that parents of SN children with a monthly family income above 15k dollars have the lowest levels of anxiety. Our results do not show any differences in parent State and Trait anxiety with different family Income levels and this evidence is also confirmed by the absence of correlation between anxiety and family income in HL and NH parent groups (Table 3 [**B**]).

We can speculate that in the participant groups the lack of differences in anxiety (S and T) based on Income level is due to the fact that risk and protective factors modulating anxiety are different, based on whether you have a NH or HL child. These results strongly support the idea that a re‐evaluation of the impact of Income on anxious symptomatology is necessary. In fact, since 2020, Covid‐19 has crucially affected the development of economies and wider society, in Italy as well as throughout the world (ISTAT, 2021). Many families have lost their jobs and have had reduced standards of living (MEF, 2021): it is clearly recognized that lower levels of household income are associated with several mental disorders (Sareen et al., 2011). Therefore, an assessment of the anxious state of parents is essential for targeted interventions especially in families with impaired children.

Concerning the Education Level of parents, Ren and colleagues (2020) highlighted that during the Covid‐19 pandemic, parents of SN children with a College education or above experienced a lower level of state anxiety than those who only reached Senior High School. Similarly, mothers of disabled children with lower educational levels had the most elevated Trait anxiety (Bumin et al., 2008). Moreover, a study conducted in Australia during the equine flu epidemic, found that minor educated groups were at greater risk of mental distress (Taylor et al., 2008). In contrast to these studies, but in line with Mappa et al. (2020), who observed that a higher educational status was associated with increased prevalence of anxiety, our results do not show significant differences regarding parent's education level. Although level of education does not seem to affect the anxiety level in adult participants, the only group that showed a significantly strong negative correlation between State Anxiety and Education Level were the parents of NH children (*r* = −0.66, *p* < 0.05). We can hypothesize that education was a protective factor against anxiety in the parent‐child dyad during the quarantine period because parents with higher educational qualifications are more likely to learn and master the skills necessary to cope with their anxiety, avoid experiencing its adverse effects and passing them on to their children. Moreover, parents of HL children, showed significative positive correlation between State anxiety and level of *Concern* about the school closures (Table 3 [**B**]).

## 5 CONCLUSION

In conclusion, the intent of the COCLOVID study was to open a small window on our collective understanding of the educational and psychological wellness of children experienced during the complex pandemic period currently being faced and which may be particularly difficult for students with hearing difficulties and their families.

The results of the present study allow us to answer initial questions as follows:

(1) In our sample participating in online education during the Covid‐19 pandemic and having hearing difficulties or being a parent of a child with hearing difficulties did not seem to affect School Wellbeing.
(2) Although much of the literature generally reported significant psychological differences between students with SNs and their peers, results of the present study do not show macro differences between hearing impaired and normal hearing students for anxiety levels experienced during the lockdown. At the same time, this lack of differentiation based on deafness was also present amongst parents who nevertheless also showed moderate anxiety symptoms. It is possible to suggest that the level of anxiety of parents may have risen due to Covid‐19 and not to their children's impairment.
(3) Normal hearing parent‐child dyad seems to show the strongest correlation in terms of parental anxiety and children's defensive attitude. Different psychological costs between children, with or without hearing impairments, can be observed in term of the relationship between defensive attitude and relationships with classmates and teachers.

## 6 LIMITATIONS

We are aware that the use of an online tool is not the optimum methodological choice available especially when the objective is the assessment of sensitive variables such as psychological ones. However, this choice was necessary to reach participants in a short period of time and during a pandemic, when face‐to‐face contacts were forbidden or severely restricted. Furthermore, although bias can affect any survey (Pierce et al., 2020), the methodology adopted in our study made it possible to avoid interpretative bias due to participants' hearing difficulties. Additionally, although the results are limited by the size of the sample observed they appear to be a relevant contribution to the debate on the impact of online education, as faced by students around the world. A final limitation of the study, shared with most existing empirical studies on Covid‐19, is the difficulty of parsing causal relationships due to collecting self‐reporting measures with no prepandemic baseline available. A future comparison with the results of an investigation undertaken in a normal educational situation with in class learning may provide support for a causal analysis and could give direction for a targeted intervention on the wellbeing of students and their families in the broader context on an effective inclusive school.

## CONFLICT OF INTEREST

The authors declare no conflict of interest.

## Supporting information

Supplementary information.
