# Supplementary material for: School wellbeing and psychological characteristics of online learning in families of children with and without hearing loss during the Covid‐19 pandemic
Source: Psychol Sch. 2022 Jul 13:10.1002/pits.22761. Online ahead of print. doi: 10.1002/pits.22761 (PMC9350277; doi:10.1002/pits.22761)
Supplement: Supplementary file 1 — Supplementary information. [file PITS-9999-0-s001.pdf]

APPENDIX

Table S1

PARENTS	Overall Sample (N=61)	Child NH (N=18)	Child HL (N=43)
Sex, n (%)			
Male	10 (16.393)	2 (11.111)	8 (18.605)
Female	51 (83.607)	16 (88.889)	35 (81.395)
Age, (μ±s.d.)	44.885 ±6.298	47.00±5.379	44±6.499
Education, n (%)			
Middle school only	9 (14.754)	0 (0.000)	9 (20.930)
High School	22 (36.066)	6 (33.333)	16 (37.209)
Undergraduate	24 (39.344)	9 (55.556)	14 (32.558)
Post-graduate	6 (9.836)	2 (11.111)	4 (9.302)
Family income, n (%)			
<36 000 €	30 (49.180)	4 (22.222)	26 (60.465)
36k – 70k €	26 (42.623)	11 (61.111)	15 (34.884)
70k – 100k €	4 (6.557)	3 (16.667)	1 (2.326)
> 100k €	1 (1.639)	0 (0.000)	1 (2.326)
Level of concern about school closure, n (%)			
Not at all	2 (3.279)	0 (0.000)	2 (4.651)
Slightly	7 (11.475)	2 (11.111)	5 (11.628)
Quite	28 (45.902)	7 (38.889)	21 (48.837)
Very	18 (29.508)	7 (38.889)	11 (25.581)
Extremely	6 (9.836)	2 (11.111)	4 (9.3.2)
Strength of home internet connection, n (%)			
Excellent	14 (22.951)	7 (38.889)	7 (16.279)
Good	25 (40.984)	8 (44.444)	17 (39.535)
Fair	21 (34.426)	3 (16.667)	18 (41.860)
Poor	1 (1.639)	0 (0.000)	1 (2.326)
General opinion about online-learning, n (%)			
Useless	1 (1.639)	0 (0.000)	1 (2.326)
Not very useful	15 (24.590)	5 (27.778)	10 (23.256)
Quite useful	33 (54.098)	11 (61.111)	22 (51.163)
Very useful	8 (13.115)	0 (0.000)	8 (18.605)
Extremely useful	4 (6.557)	2 (11.111)	2 (4.651)
Feel that their child is wasting their time, n (%)			
Yes	11 (18.033)	2 (11.111)	9 (20.930)
No	42 (68.852)	13 (72.222)	29 (67.442)
Don't know	8 (13.115)	3 (16.667)	5 (11.628)
Consider lessons online to be an advantage for your child, n (%)			
Yes	38 (62.295)	12 (66.667)	26 (60.465)
No	15 (24.590)	5 (27.778)	10 (23.256)
Don't know	8 (13.115)	1 (5.556)	7 (16.279)
Level of child's hearing capacity (see typologies*), n (%)			
NH	18 (29.508)	18 (100.000)	0 (0.000)
HA	10 (16.393)	0 (0.000)	10 (23.256)
BIM	9 (14.754)	0 (0.000)	9 (20.930)
UCI	13 (21.311)	0 (0.000)	13 (30.233)
BCI	11 (18.033)	0 (0.000)	11 (25.581)
Need for parental presence during online lessons, n (%)			
Yes	13 (21.311)	1 (5.556)	12 (27.907)
No	48 (78.689)	17 (94.444)	31 (72.093)
Opinion about child's understanding of what teachers are saying during online lessons, n (%)			
No understanding	1 (1.639)	1 (5.556)	0 (0.000)
Some Understanding	7 (11.475)	0 (0.000)	7 (16.279)
Fair Understanding	21 (34.426)	3 (16.667)	18 (41.860)
Good Understanding	24 (39.344)	9 (50.000)	15 (34.884)
Extremely Good Understanding	8 (13.115)	5 (27.778)	3 (6.977)
Teachers use additional materials/tools for your son/daughter, n (%)			
Yes	29 (47.541)	8 (44.444)	21 (48.837)
No	32 (52.459)	10 (55.556)	22 (51.163)
How do you feel your son/daughter appears following online lessons, n (%)			
Satisfied	17 (27.869)	5 (27.778)	12(27.907)
Tired	26 (42.623)	11 (61.111)	15 (34.884)
Nervous	9 (14.754)	3 (16.667)	6 (13.953)
He/she reports headaches	6 (9.836)	1 (5.556)	5 (11.628)
He/she reports burning eyes	1 (1.639)	1 (5.556)	0 (0.000)
I don't notice anything in particular	15 (24.590)	2 (11.111)	13 (30.233)

STUDENTS	Overall Sample (N=61)	Students NH (N=18)	Student HL (N=43)
Sex, n (%)			
Male	25 (40.984)	5 (27.778)	20 (46.512)
Female	36 (59.016)	13(72.222)	23 (53.488)
Age, μ±(S.D.)	11 (±1.732)	12.94 (±2.838)	11 (±1.732)
Education, n (%)			
Elementary school- 3rd	9 (14.754)	1 (5.556)	8 (18.605)
Elementary school- 4th	78 (13.115)	4 (22.222)	4 (9.302)
Elementary school- 5th	4 (6.557)	2 (11.111)	2 (4.651)
Middle school-6th	7 (11.475)	1 (5.556)	6 (13.953)
Middle school- 7th	11 (18.033)	1 (5.556)	10 (23.256)
Middle school- 8th	6 (9.836)	2 (11.111)	4 (9.3.2)
High school-9th	2 (3.279)	2 (11.111)	0 (0.000)
High school-10th	4 (6.557)	1 (5.556)	3 (6.977)
High school-11th	6 (9.836)	3 (16.667)	3 (6.977)
High school-12h	3 (4.918)	1 (5.556)	2 (4.651)
High school-13th	1 (1.639)	0 (0.000)	1 (2.326)
Currently taking lessons online			
Yes	57 (93.443)	16 (88.889)	41 (95.349)
No	4 (6.557)	2 (11.111)	2 (4.651)
Usual mode of connection to online lessons			
Mobile phone	14 (22.951)	3 (16.667)	11 (25.581)
Personal computer (PC)	39 (63.934)	11 (61.111)	28 (65.115)
Tablet	12 (19.672)	2 (11.111)	10 (23.256)
No Answer	4 (6.557)	2 (11.111)	2 (4.651)
Presence of a fixed workstation at home to follow online lessons, n (%)			
Yes, in my bedroom	26 (42.6223)	9 (50.000)	17 (39.535)
Yes, in my living room	11 (18.033)	3 (16.667)	8 (18.605)
Yes, in my studio/office	4 (6.557)	2 (11.111)	2 (4.651)
Yes, in my kitchen	8 (13.115)	2 (11.111)	6 (13.953)
I do not have a fixed location	8 (13.115)	0 (0.000)	8 (18.605)
No answer	4 (6.557)	2(11.111)	2 (4.651)
When you are connected for online lessons, mostly, n (%)			
You are alone	42 (68.852)	13 (72.222)	29 (67.442)
You are with your mother	12 (19.672)	2 (11.111)	10 (23.256)
You are with your father	6 (9.836)	0 (0.000)	6 (13.953)
You are with your brother(s) and/or sister(s)	1 (1.639)	1 (5.556)	0 (0.000)
No answer	4 (6.557)	2 (11.111)	2 (4.651)
Program/platform used by the school to organize online lessons, n (%)			
Classroom	25 (40.984)	7 (38.889)	18 (41.860)
We school	9 (14.754)	0 (0.000)	9 (20.930)
Moodle	0 (0.000)	0 (0.000)	0 (0.000)
Edmodo	3 (4.918)	1 (5.556)	2 (4.651)
Skype	3 (4.918)	0 (0.000)	3 (6.977)
WhatsApp	2 (3.279)	0 (0.000)	2 (4.651)
Other	31 (50.820)	9 (50.000)	22 (51.163)
No answer	4 (6.557)	2 (11.111)	2 (4.651)
Online lessons are listened to via, n (%)			
Speakers (on the PC or connected to the device used)	33 (54.098)	11 (61.111)	22 (51.163)
Earphones	22 (36.066)	5 (27.778)	17 (39.535)
Bluetooth connection system	3 (4.918)	0 (0.000)	3 (6.977)
FM system	2 (3.279)	0 (0.000)	2 (4.651)
No answer	6 (9.836)	2 (11.111)	4 (9.302)
During lessons online, the webcam is active, n (%)			
Always	38 (62.295)	11 (61.111)	27 (62.791)
Never	1 (1.639)	1 (5.556)	0 (0.000)
Only for some subjects	18 (29.508)	4 (22.222)	14 (32.558)
No answer	4 (6.557)	2 (11.111)	2 (4.651)
How enjoyable it is to have lessons online, n (%)			
Not at all	5 (8.197)	2 (11.111)	3 (6.977)
Slightly enjoyable	11 (18.033)	4 (22.222)	7 (16.279)
Quite enjoyable	30 (49.180)	9 (50.000)	21 (48.837)
Very enjoyable	7 (11.475)	1 (5.556)	6 (13.953)
Extremely enjoyable	4 (6.557)	0 (0.000)	4 (9.302)
No answer	4 (6.557)	2 (11.111)	2 (4.651)

Audio clarity during online lessons, n (%)			
Not at all	1 (1.639)	0 (0.000)	1 (2.326)
Slightly clear	7 (11.475)	3 (16.667)	4 (9.302)
Quite clear	35 (57.377)	8 (44.444)	27 (62.791)
Very clear	10 (16.393)	3 (16.667)	7 (16.279)
Extremely clear	4 (6.557)	2 (11.111)	2 (4.651)
No answer	4 (6.557)	2 (11.111)	2 (4.651)
Which voice is best understood online, n (%)			
Male	2 (3.279)	0 (0.000)	2 (4.651)
Female	6 (9.836)	2 (11.111)	4 (9.302)
Both	47 (77.049)	14 (77.778)	33 (76.744)
None	2 (3.279)	0 (0.000)	2 (4.651)
No answer	4 (6.557)	2 (11.111)	2 (4.651)
There is something specific that makes it difficult to understand what the teacher is saying, n (%)			
Speed of speech	20 (32.787)	5 (27.778)	15 (34.884)
Loud volume voice	7 (11.475)	2 (11.111)	5 (11.628)
Low volume voice	20 (32.787)	7 (38.889)	13 (30.233)
Teacher's beard	0 (0.000)	0 (0.000)	0 (0.000)
Head movements	1 (1.639)	1 (5.556)	0 (0.000)
Nothing	16 (26.230)	4 (22.222)	12 (27.907)
Other	8 (13.115)	4 (22.222)	4 (9.302)
No answer	4 (6.557)	2 (11.111)	2 (4.651)
Opinion about online lessons, n (%)			
Boring	14 (22.951)	5 (27.778)	9 (20.930)
Interesting	11 (18.033)	3 (16.667)	8 (18.605)
Normal	28 (45.902)	8 (44.444)	20 (46.512)
Tiring	14 (22.951)	6 (33.333)	8 (18.605)
Difficult to follow	8 (13.115)	0 (0.000)	8 (18.605)
Funny	6 (9.836)	1 (5.556)	5 (11.628)
No answer	4 (6.557)	2 (11.111)	2 (4.651)
Reason why you are not currently taking online lessons, n (%)			
Lack of internet connection at home	0 (0.000)	0 (0.000)	0 (0.000)
Lack of PC/tablet/cell phone to connect with	0 (0.000)	0 (0.000)	0 (0.000)
Lack of desire	0 (0.000)	0 (0.000)	0 (0.000)
Too much personal difficulty in following online lessons	0 (0.000)	0 (0.000)	0 (0.000)
My teacher (and/or support teacher) send homework directly	4 (6.557)	2 (11.111)	2 (4.651)
No answer	57 (93.443)	16 (88.888)	41 (95.349)

Table S1- Socio-demographic characteristics and synthesis of the responses to questions about online learning of the participants (Parents and Students) and their distribution based on hearing characteristics (Normal hearing-NH; Hearing loss-HL).

Table S3

Groups		UCI	UCI	UCI	UCI	UCI	BIM	BIM	BIM	BIM	BIM	HA	HA	HA	HA	HA	BCI	BCI	BCI	BCI	BCI	NH	NH	NH	NH	NH
	QBS scales*	*GBS*	*RWC*	*RWT*	*EA*	*SE*	*GBS*	*RWC*	*RWT*	*EA*	*SE*	*GBS*	*RWC*	*RWT*	*EA*	*SE*	*GBS*	*RWC*	*RWT*	*EA*	*SE*	*GBS*	*RWC*	*RWT*	*EA*	*SE*
		μ 58.857	*μ 48.429*	*μ 39.571*	*μ58.571*	*μ 56.286*	*μ 48.667*	*μ 49*	*μ 50.556*	*μ 55.111*	*μ 44.444*	*μ 50.875*	*μ 42.875*	*μ 37.125*	*μ 40.875*	*μ 38.625*	*μ 52.9*	*μ 48.1*	*μ 42.8*	*μ 49.8*	*μ 47.4*	*μ 50.455*	*μ 49.364*	*μ 49.909*	*μ 54.455*	*μ 49.273*
**UCI**	*GBS*		0.020567	0.000003	0.938825	0.519248	0.091573	0.101604	0.152566	0.491879	0.016402	0.16427	0.00738	0.000193	0.002384	0.000561	0.294927	0.076518	0.007258	0.127238	0.05938	0.151606	0.111584	0.129242	0.431402	0.110037
**UCI**	*RWC*	0.020567		0.04052	0.024117	0.08708	0.96126	0.913677	0.723003	0.269075	0.464608	0.685063	0.319728	0.052774	0.188773	0.092483	0.457301	0.946504	0.322792	0.81518	0.844658	0.734073	0.872034	0.802501	0.31722	0.878769
**UCI**	*RWT*	0.000003	0.04052		0.000005	0.000068	0.119658	0.109246	0.06924	0.009391	0.383379	0.062148	0.544714	0.640536	0.789871	0.846603	0.026888	0.136458	0.537501	0.087322	0.166247	0.070801	0.100366	0.085418	0.012937	0.101494
**UCI**	*EA*	0.938825	0.024117	0.000005		0.539194	0.099904	0.110537	0.162444	0.508606	0.018451	0.17371	0.008412	0.000232	0.002772	0.000666	0.309422	0.083881	0.008285	0.137237	0.065411	0.162187	0.120825	0.138971	0.449748	0.119393
**UCI**	*SE*	0.519248	0.08708	0.000068	0.539194		0.206672	0.225241	0.31401	0.810244	0.050168	0.332503	0.025895	0.00114	0.010059	0.002915	0.534696	0.177933	0.025512	0.271277	0.144394	0.312815	0.243638	0.274822	0.726875	0.240615
**BIM**	*GBS*	0.091573	0.96126	0.119658	0.099904	0.206672		0.928655	0.676597	0.160484	0.321742	0.71287	0.308765	0.04941	0.180177	0.087224	0.478966	0.914394	0.309833	0.844172	0.817405	0.762638	0.900989	0.832287	0.334245	0.908379
**BIM**	*RWC*	0.101604	0.913677	0.109246	0.110537	0.225241	0.928655		0.727852	0.181124	0.29461	0.753216	0.288679	0.044232	0.16601	0.079007	0.51117	0.870465	0.288565	0.88532	0.777013	0.804205	0.945449	0.875558	0.359965	0.955606
**BIM**	*RWT*	0.152566	0.723003	0.06924	0.162444	0.31401	0.676597	0.727852		0.284209	0.184355	0.947988	0.202961	0.02566	0.10963	0.048371	0.654574	0.683951	0.200508	0.89194	0.602488	0.983615	0.835358	0.9023	0.47427	0.82692
**BIM**	*EA*	0.491879	0.269075	0.009391	0.508606	0.810244	0.160484	0.181124	0.284209		0.017484	0.436645	0.042603	0.002378	0.017887	0.005699	0.673071	0.247951	0.041999	0.364329	0.204917	0.414423	0.330478	0.368652	0.893269	0.326712
**BIM**	*SE*	0.016402	0.464608	0.383379	0.018451	0.050168	0.321742	0.29461	0.184355	0.017484		0.287788	0.748354	0.203202	0.512649	0.306416	0.162087	0.484894	0.753834	0.364991	0.545889	0.315765	0.401233	0.359067	0.097682	0.404425
**HA**	*GBS*	0.16427	0.685063	0.062148	0.17371	0.332503	0.71287	0.753216	0.947988	0.436645	0.287788		0.081055	0.00155	0.027233	0.005651	0.678955	0.646941	0.183986	0.853095	0.567724	0.936696	0.796725	0.86087	0.494048	0.786641
**HA**	*RWC*	0.00738	0.319728	0.544714	0.008412	0.025895	0.308765	0.288679	0.202961	0.042603	0.748354	0.081055		0.182629	0.616528	0.318503	0.097166	0.336453	0.987864	0.243435	0.386859	0.206733	0.271058	0.239178	0.054824	0.27321
**HA**	*RWT*	0.000193	0.052774	0.640536	0.000232	0.00114	0.04941	0.044232	0.02566	0.002378	0.203202	0.00155	0.182629		0.365941	0.686978	0.008269	0.058244	0.309113	0.033804	0.074209	0.026318	0.039939	0.032954	0.00347	0.040469
**HA**	*EA*	0.002384	0.188773	0.789871	0.002772	0.010059	0.180177	0.16601	0.10963	0.017887	0.512649	0.027233	0.616528	0.365941		0.573023	0.046525	0.202298	0.693976	0.135501	0.241177	0.111958	0.153727	0.132737	0.023948	0.155336
**HA**	*SE*	0.000561	0.092483	0.846603	0.000666	0.002915	0.087224	0.079007	0.048371	0.005699	0.306416	0.005651	0.318503	0.686978	0.573023		0.017551	0.100916	0.443334	0.062031	0.125126	0.049531	0.072041	0.060577	0.008027	0.072947
**BCI**	*GBS*	0.294927	0.457301	0.026888	0.309422	0.534696	0.478966	0.51117	0.654574	0.673071	0.162087	0.678955	0.097166	0.008269	0.046525	0.017551		0.301199	0.025539	0.478673	0.237209	0.654291	0.542699	0.593978	0.750662	0.537573
**BCI**	*RWC*	0.076518	0.946504	0.136458	0.083881	0.177933	0.914394	0.870465	0.683951	0.247951	0.484894	0.646941	0.336453	0.058244	0.202298	0.100916	0.301199		0.210878	0.703643	0.850853	0.694787	0.829468	0.761622	0.293584	0.83797
**BCI**	*RWT*	0.007258	0.322792	0.537501	0.008285	0.025512	0.309833	0.288565	0.200508	0.041999	0.753834	0.183986	0.987864	0.309113	0.693976	0.443334	0.025539	0.210878		0.122839	0.265678	0.204523	0.26934	0.236891	0.0541	0.272254
**BCI**	*EA*	0.127238	0.81518	0.087322	0.137237	0.271277	0.844172	0.88532	0.89194	0.364329	0.364991	0.853095	0.243435	0.033804	0.135501	0.062031	0.478673	0.703643	0.122839		0.595648	0.901044	0.928966	0.982289	0.421722	0.920326
**BCI**	*SE*	0.05938	0.844658	0.166247	0.065411	0.144394	0.817405	0.777013	0.602488	0.204917	0.545889	0.567724	0.386859	0.074209	0.241177	0.125126	0.237209	0.850853	0.265678	0.595648		0.612599	0.74012	0.675862	0.244997	0.746457
**NH**	*GBS*	0.151606	0.734073	0.070801	0.162187	0.312815	0.762638	0.804205	0.983615	0.414423	0.315765	0.936696	0.206733	0.026318	0.111958	0.049531	0.654291	0.694787	0.204523	0.901044	0.612599		0.79392	0.883513	0.348375	0.783531
**NH**	*RWC*	0.111584	0.872034	0.100366	0.120825	0.243638	0.900989	0.945449	0.835358	0.330478	0.401233	0.796725	0.271058	0.039939	0.153727	0.072041	0.542699	0.829468	0.26934	0.928966	0.74012	0.79392		0.891549	0.254513	0.980532
**NH**	*RWT*	0.129242	0.802501	0.085418	0.138971	0.274822	0.832287	0.875558	0.9023	0.368652	0.359067	0.86087	0.239178	0.032954	0.132737	0.060577	0.593978	0.761622	0.236891	0.982289	0.675862	0.883513	0.891549		0.295709	0.878943
**NH**	*EA*	0.431402	0.31722	0.012937	0.449748	0.726875	0.334245	0.359965	0.47427	0.893269	0.097682	0.494048	0.054824	0.00347	0.023948	0.008027	0.750662	0.293584	0.0541	0.421722	0.244997	0.348375	0.254513	0.295709		0.251265
**NH**	*SE*	0.110037	0.878769	0.101494	0.119393	0.240615	0.908379	0.955606	0.82692	0.326712	0.404425	0.786641	0.27321	0.040469	0.155336	0.072947	0.537573	0.83797	0.272254	0.920326	0.746457	0.783531	0.980532	0.878943	0.251265	

Table S3 - Differences in School Wellbeing in Junior Students Hearing Groups.
The Table shows the post hoc results performed on the significant interaction between the factors QBS scales and Hearing Groups
Significant pairwise differences are indicated in red ($p \leq 0.05$).
*=abbreviations of QBS child's scales: *GBS-Gratification obtained by school results*; *RWT-Relationship with teachers*; *RWC-Relationship with classmates*; *EA-Emotional attitude towards school*; *SE-Self Efficacy*.
